# Research on Finger Pressure Tactile Sensor with Square Hole Structure Based on Fiber Bragg Grating

**DOI:** 10.3390/s23156897

**Published:** 2023-08-03

**Authors:** Guan Lu, Shiwen Fu, Tianyu Zhu, Yiming Xu

**Affiliations:** 1 School of Mechanical Engineering, Nantong University, Nantong 226019, China; luguan@ntu.edu.cn (G.L.); 2109310002@stmail.ntu.edu.cn (S.F.); 18861351101@163.com (T.Z.); 2 School of Electrical Engineering, Nantong University, Nantong 226019, China

**Keywords:** pressure tactile sensing, fiber grating, robot finger

## Abstract

Aiming at the problems of lateral force interference and non-uniform strain of robot fingers in the process of pressure tactile sensing, a flexible tactile sensor with a square hole structure based on fiber Bragg grating (FBG) is proposed in this paper. Firstly, the optimal embedding depth of the FBG in the sensor matrix model was determined by finite element simulation. Secondly, according to the size of the finger knuckle and the simulation analysis based on the pressure tactile sensor element for the robot finger, the square hole structure was designed, and the overall dimensions of the sensing element and size of the square hole were determined. Thirdly, the FBG was embedded in the polydimethylsiloxane (PDMS) elastic matrix to make a sensor model, and the tactile sensor was fabricated. Finally, the FBG pressure tactile sensing system platform was built by using optical fiber sensing technology, and the experiment of the FBG tactile sensor was completed through the sensing system platform. Experimental results show that the tactile sensor designed in this paper has good repeatability and creep resistance. The sensitivity is 8.85 pm/N, and the resolution is 0.2 N. The loading sensitivity based on the robot finger is 27.3 pm/N, the goodness of fit is 0.996, and the average value of interference in the sensing process is 7.63%, which is lower than the solid structure sensor. These results verify that the sensor can effectively reduce the lateral force interference and solve the problem of non-uniform strain and has high fit with fingers, which has a certain application value for the research of robot pressure tactile intelligent perception.

## 1. Introduction

As one of the most important functions of intelligent robot fingers, tactile perception has always been the focus of robot intelligent perception research. The robot’s tactile perception of the outside world mainly depends on fingers, bionic skin, and other aspects. The tactile research based on robotic fingers can help the robot more actively perceive the interaction between the external environment and objects and carry out timely judgment and feedback. At the same time, multiple joints can also complete various complex operations. Therefore, the robot’s cognition of external information is closely related to the tactile perception ability of robot fingers.

Common tactile sensors applied to robot fingers include piezoresistive sensors, piezoelectric sensors, and capacitive sensors. With an increasing number of robots applied in complex working environments, traditional electrical sensors are inevitably affected by signal noise and electromagnetic interference [1,2,3]. On the other hand, the packaging problem of tactile sensors cannot be ignored. At present, the research of tactile sensors mostly adopts flexible matrices. Among them, the physical characteristics of polydimethylsiloxane (PDMS) are close to the human skin. It is often used to make bionic skin, which is suitable for making a tactile sensor matrix [4,5,6,7,8]. However, the existing piezoresistive, piezoelectric, and capacitive sensors have poor compatibility with flexible materials [9], which has a certain impact on the measurement results.

In recent years, with the development of optical fiber sensing technology, FBG has been widely used in the sensing field. Because of its small size, easy packaging, simple structure, flexibility, ease of embedding in materials, and anti-electromagnetic interference, FBG has gradually been used as a new type of flexible tactile sensor by scholars at home and abroad [10,11,12,13,14]. Hao et al. [15] pasted FBG on the inner wall of a hollow glass tube to make an optical fiber F-P tactile sensor with high sensitivity for surgery, but its sensitivity is better only in a small range. Saccomandi et al. [16] designed a PDMS matrix with grooves and embedded FBG engraved with multiple gratings into the matrix to form an array tactile sensor. The sensor has good linearity but low sensitivity. Jiang et al. [17] installed optical fiber whisker arrays on both sides of a robot, which can recognize the contour shape and surface texture features of the contact object. Samuel et al. [3] embedded the FBG in silicone to detect the pressure on the vertical surface and identified cracks greater than 0.3 mm through 3D mapping of fatigue cracks. Qian et al. [18] designed and fabricated a distributed flexible tactile sensor based on two-dimensional fiber Bragg grating (FBG) sensing, which has high linearity and sensitivity. Qian et al. [19] conducted an experimental study on material recognition of an FBG flexible tactile sensor applied to electronic skin, which can realize the perception of more modal information of objects in electronic skin. Feng et al. [20] have designed a flexible wearable smart sleeve with four heads of FBG and spandex polyurethane fibers (SPFs), which can be used to develop FBG sensing systems for monitoring joint movements for different human sizes online. In summary, the research on the FBG tactile sensor mainly focuses on the sensing method and structural design, and there is little research on the problems of lateral force interference and non-uniform strain in the process of sensing and monitoring. The lateral force interference refers to the lateral interference of the tangential force between the contact object and the matrix material on the sensor, which affects the measurement accuracy of pressure sensing. The non-uniformity of strain means that the non-uniform distribution of strain value leads to spectral distortion when the grating section embedded in the matrix material is subjected to external load, and better strain uniformity can effectively increase the sensitivity of pressure sensing.

Although the shear force is very important in some applications, the tactile sensor in this paper is for a humanoid finger robot hand, and due to the wrapping of the matrix material, the large shear force causes the distortion of the FBG reflection spectrum and affects the accuracy of the signal output. Therefore, aiming at this problem, we studied how to effectively reduce the interference of the lateral force and enhance the sensitivity of pressure perception.

At present, there are few studies in this direction. Heo et al. [21] conducted a part of the research on non-uniform variation. His team encapsulated FBG into a disc made of PDMS and increased the resolution of the sensor by adding bosses on the disc and circular holes on the lower surface. In 2017, aiming at the problem of lateral force interference, Jiang et al. [22] made a sensing array with the arch bridge structure as a single node, and the error of the sensing force value was 0.038 N, which effectively reduced the influence of lateral force on accuracy. Qian et al. [23] used a BP neural network and an RBF neural network to conduct neural network-based fiber grating tactile signal decoupling research.

Aiming at the above problems, in this study we designed a finger pressure tactile sensor which can effectively reduce the lateral force interference, solved the non-uniform strain problem with the interference of external force, and carried out a simulation and performance experiment on the fabricated FBG pressure tactile sensor. Using the square hole structure, the pressure in the contact process can be effectively transformed into the axial tensile deformation of fiber Bragg grating. Compared with the existing studies, the pressure tactile sensor designed in this study can be better applied to the humanoid finger robot hand. At the same time, the pressure tactile sensing experiment was carried out on the robot finger, which proved its effectiveness: the FBG pressure tactile sensing element can be used in the robot finger, and can effectively realize the tactile sensing of external pressure. The research in this paper can provide a certain theoretical and experimental basis for the research of robot tactile sensing, and promote the research and application of the FBG tactile sensor in robot intelligent sensing.

## 2. FBG Pressure Tactile Sensing Principle

**Figure 1:**
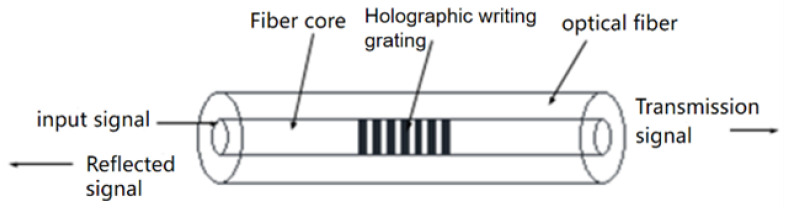
Principle of fiber grating sensing.

Fiber Bragg grating is a phase grating formed in the fiber core by the interaction between germanium ions in the fiber core and external incident photons. Figure 1 is the structural diagram of FBG. The reflection and transmission characteristics are shown in the figure. When broadband light wave passes through fiber grating, reflection and transmission occur due to different refractive indices. The incident light that satisfies the Bragg reflection condition is reflected, and the transmission spectrum of the transmitted light has a concave peak, which reflects the light of a specific wavelength. Bragg reflection conditions are [24]:

(1) $$\lambda_{B}=2n_{eff}\Lambda$$

Among them, $\lambda_{B}$ is the reflection wavelength of the FBG, $n_{eff}$ is the effective refractive index of the fiber core, and Λ is the grating pitch. It can be seen from the formula that when the effective refractive index of the grating core or the grating pitch changes, the reflection center wavelength of the FBG also changes.

Many external factors, such as strain, temperature, slip, and vibration, cause the reflection wavelength to change, among which strain and temperature are the most influential factors. Strain is the most direct factor affecting the change in Bragg grating center wavelength. Under the condition of constant temperature (ΔT = 0), the Bragg grating shift caused by strain can be expressed as:

(2) $$\Delta \lambda_{B}=2n_{eff}\Delta \Lambda +2\Delta n_{eff}\Lambda$$

Among them, $\Delta \lambda_{B}$ represents the geometric deformation of the fiber grating under external force conditions, and $\Delta n_{eff}$ represents the elastic–optical effect. Expressed by differential formula, the above formula is:

(3) $$\frac{\Delta \lambda_{B}}{\lambda_{B}}=\left(\frac{1}{n_{eff}}\frac{\partial n_{eff}}{\partial \epsilon }+\frac{1}{\Lambda }\frac{\partial \Lambda }{\partial \epsilon }\right)$$

Equation (3) is the strain sensing model of the FBG at constant temperature, which shows that it is feasible to test the tactile sensing signal with FBG in theory.

## 3. Design and Simulation of FBG Pressure Tactile Sensing Element

### 3.1. Sensing Element Modeling and Grating Embedding Depth Analysis

**Figure 2:**
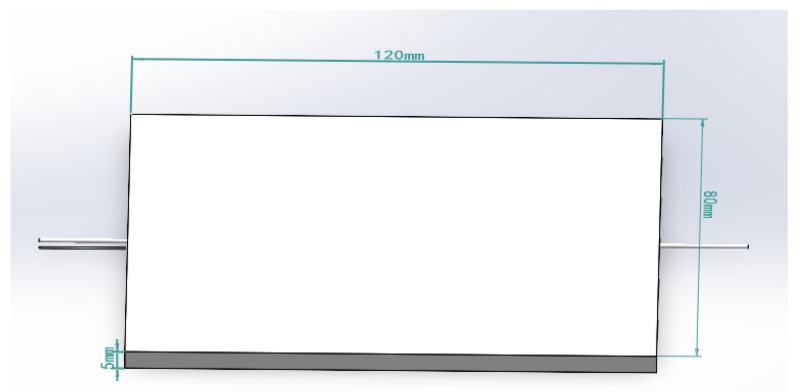
PDMS matrix model with embedded fiber Bragg grating.

The matrix of the flexible tactile sensor based on FBG adopts PDMS material with similar mechanical properties to human skin. When the matrix is subjected to external pressure, the internal optical fiber deforms, resulting in the central wavelength shift of the grating. The matrix model was established in the finite element analysis software ANSYS; the size is 120 mm × 80 mm × 5 mm, and an optical fiber is embedded inside, as shown in Figure 2. The sensor flexible packaging material PDMS selected in this study is one of the commonly used packaging materials for artificial skin. This study utilized a universally applicable polydimethylsiloxane (PDMS) material and employed an encapsulation structure with uniform density. The strategy of structural modification was employed to manipulate the strain distribution of the sensing element. Finite element analysis was conducted on the sensing element, incorporating the PDMS material parameters derived from experimental measurements. The matrix material is Mooney–Rivlin, a hyperelastic material, with an elastic modulus E = 9.2 Mpa and a Poisson’s ratio of 0.49. The material of the optical fiber is germanium-doped quartz with an elastic modulus of 730 Kpa. Solid186 element was selected for grid division of the matrix model, and the meshing density is 0.5 mm. The lower surface of the matrix is fixed, cannot move in the vertical direction (*z* direction), and can expand and contract freely in the horizontal directions (*x* direction and *y* direction).

**Figure 3:**
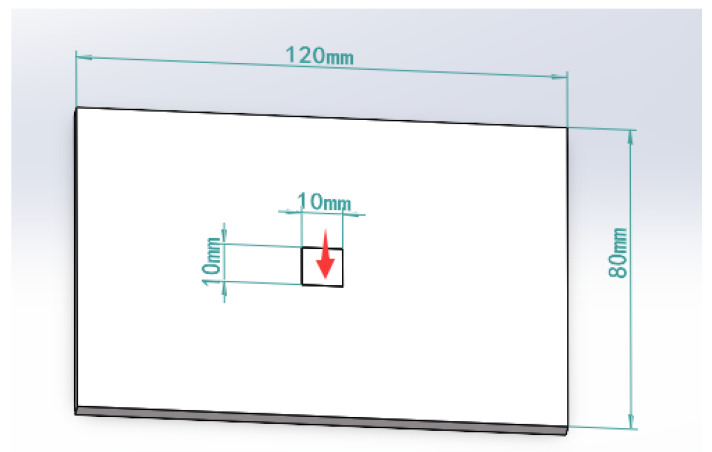
Matrix model with loading area of 1 cm${}^{2}$.

**Figure 4:**
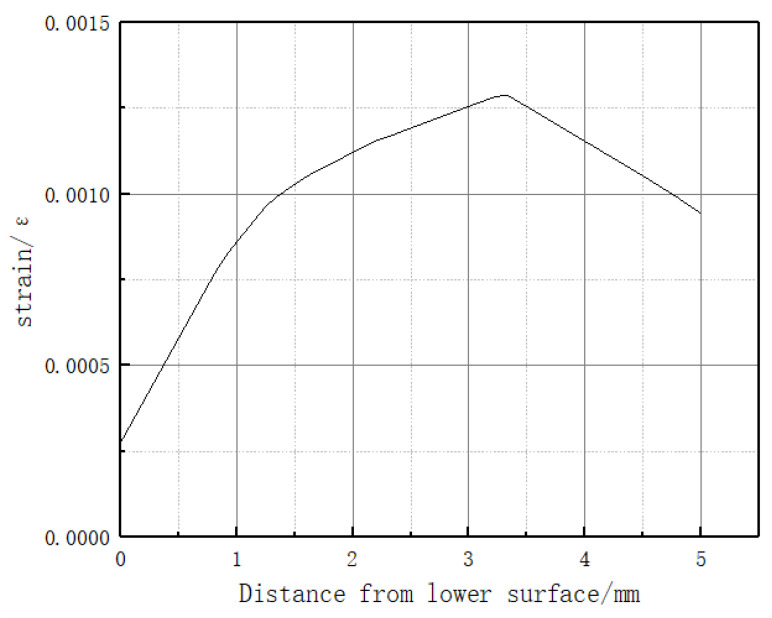
Strain on vertical path.

**Figure 5:**
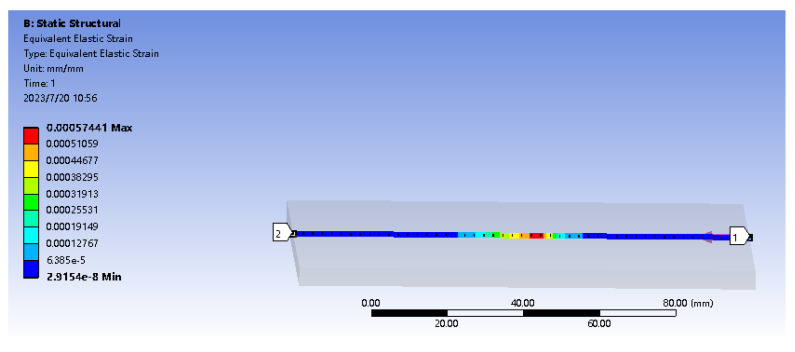
Strain simulation diagram along the *x*-axis path.

**Figure 6:**
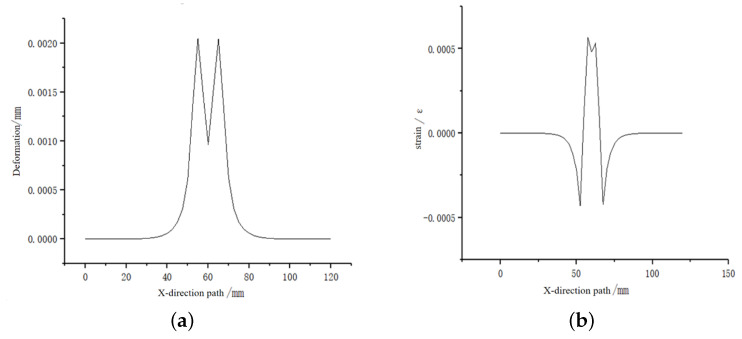
Deformation and strain diagram along the *x*-axis path under 2 N load conditions. (**a**) Path deformation graph. (**b**) Path axial strain diagram.

In the actual situation, the tactile sensor is rarely subjected to the force concentrated at one point. Therefore, a small area of uniform load loading simulation is carried out on the sensor matrix: a vertical pressure of 2 N in the *Z*-axis direction is applied on the substrate, and the loading area is 1 cm${}^{2}$. As shown in Figure 3, a path is defined along the *Z* direction, starting from (60, 40, and 0 mm) and ending at (60, 40, and 5 mm) to observe the strain on the path. As shown in Figure 4, there is a maximum strain of 1300 microstrain at the embedding depth of 1.7 mm; the more considerable strain value exists in the embedding depth range of 1–2 mm. In order to analyze the deformation and strain range of optical fiber at the optimal embedding depth of 1.7 mm under the influence of force, a path in the *X* direction is defined. As shown in Figure 5, the starting point is (0, 40, and 3.3 mm), and the endpoint is (120, 40, and 3.3 mm). The diagram of strain and deformation on the path is shown in Figure 6, and the following conclusions can be obtained:

a. Under the effect of uniform load, the deformation at the edge of the loading surface is the largest, and the deformation is reduced as it is closer to the center of the larger deformation area;

b. The axial strain on this path has a large non-uniform strain value at the edge and center of the loaded area;

c. The embedding depth directly affects the strain transfer efficiency of FBG, so as to change the central wavelength shift.

In conclusion, the simulation experiment shows that the maximum strain measured when the embedding depth of FBG is about 3/10 of the element thickness from the upper surface of the sensing element; when the embedding depth of FBG is in the range of 1/5–2/5 element thickness, the average strain on the path measured by the grating is larger.

### 3.2. Design of Pressure Tactile Sensor Element Based on the Robot Finger

**Figure 7:**
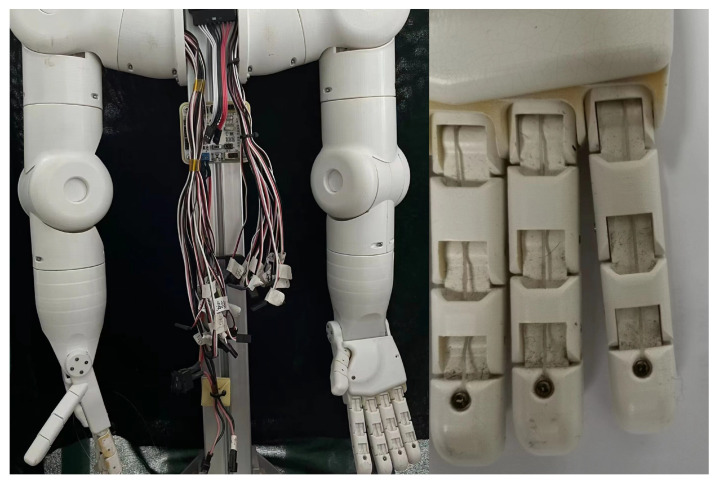
Intelligent massage robot arm and its humanoid fingers.

**Figure 8:**
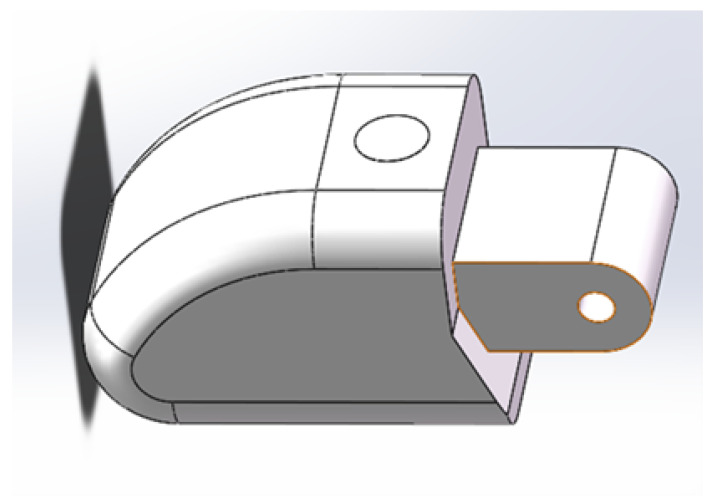
Model of robot knuckles.

Generally speaking, a dexterous mechanical finger consists of three joints. Take the dual-arm robot as an example for research, Figure 7 shows the human body massage dual-arm robot and its single finger used in the experiment. In the actual sensing process, in order to avoid the fiber breakage caused by finger bending, three FBGs can be combined and connected in series on a single fiber and fixed on the three knuckles of the finger by wavelength division multiplexing (WDM). Among the three knuckles, the joint at the end of the finger is the joint that contacts with external objects most frequently. Therefore, we selected the end knuckle as an example for research. As shown in Figure 7, the joint at the end of the finger is designed to be smooth and curved, which is more convenient for touching the object; when the manipulator grabs or presses the object, the inner surface of the joint at the end of the finger is the main contact surface. A three-dimensional model was established according to the end joint of the mechanical finger, as shown in Figure 8: the fingertips and both sides of the robot fingers have smooth rounded corners, which is not conducive to the installation of sensors, and there is a smooth plane at the fingertips, which is about 7 mm × 9 mm and suitable for arranging sensors.

**Figure 9:**
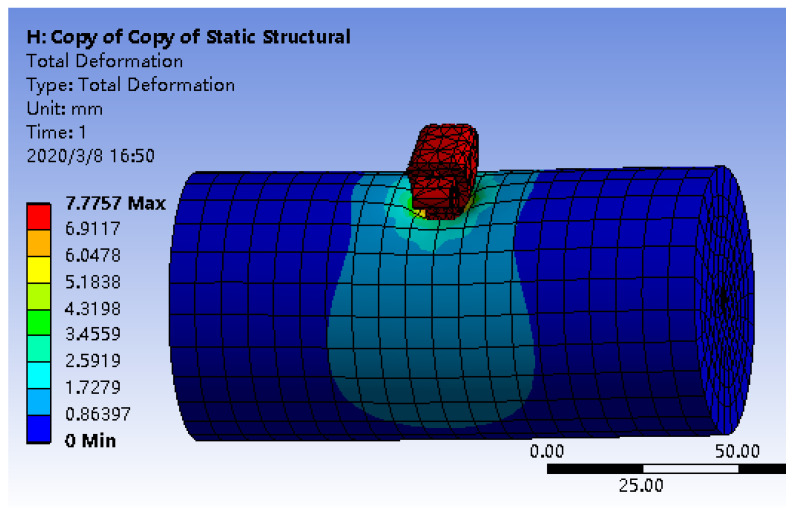
Simulation diagram of human arm under the effect of robot finger pressing.

According to medical data, the pressure that human skin can withstand is about 5 N. We used ANSYS to simulate the human arm pressed by the finger end joint, as shown in Figure 9: the normal surface of the knuckle applies a positive pressure of 2 N along the vertical direction of the arm; without the influence of bone, the arm muscle deforms under the pressure of the finger, and the maximum deformation value reaches 7.88 mm. The plane on the knuckles is the main contact plane when pressing. When the finger presses the arm to the maximum deformation, the contact plane changes slightly. According to the actual size of the end knuckle of the robot finger and the characteristics of the sensor packaging material, the overall dimensions of the tactile sensor structure were determined as 7 mm × 9 mm × 5 mm.

Based on the above size and load requirements, a new pressure tactile sensing element with holes was designed in this study, which can effectively solve the problem of lateral force interference in the process of tactile sensing and make the strain measured on the grating segment uniform.

**Figure 10:**
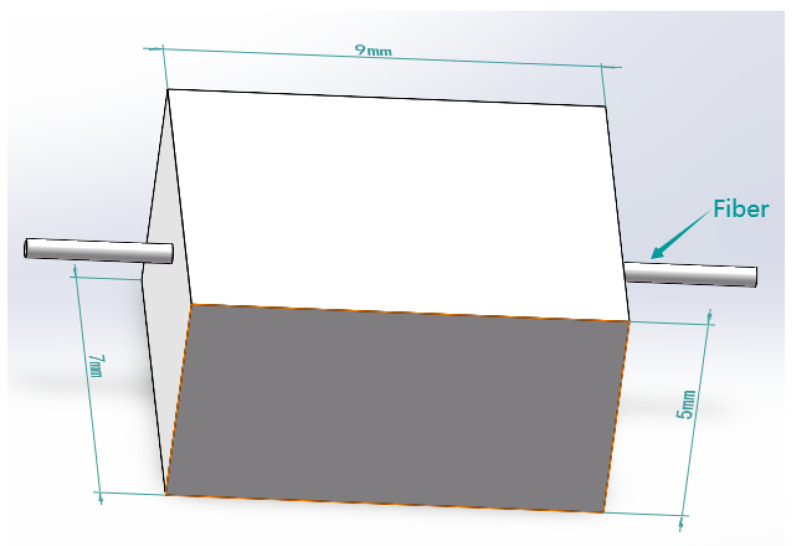
Model of the solid sensor element.

**Figure 11:**
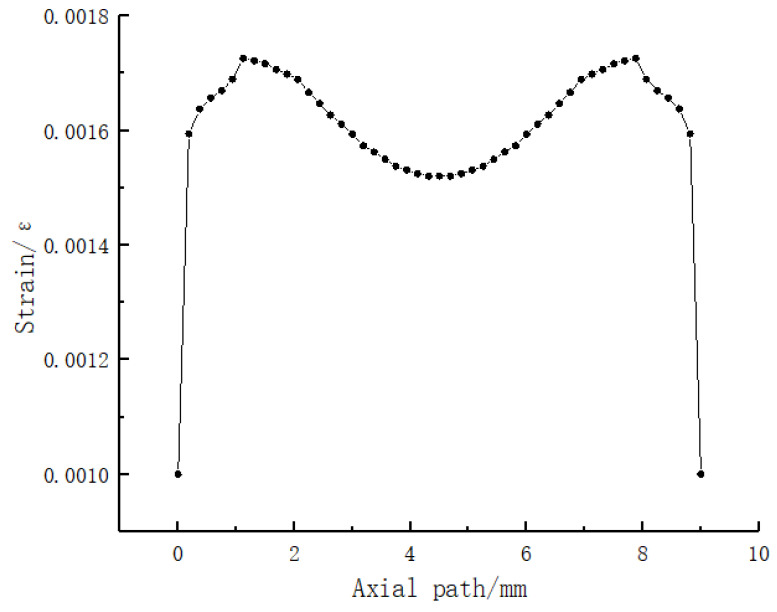
Strain variation diagram of fiber Bragg grating under 2 N uniformly distributed load conditions.

**Figure 12:**
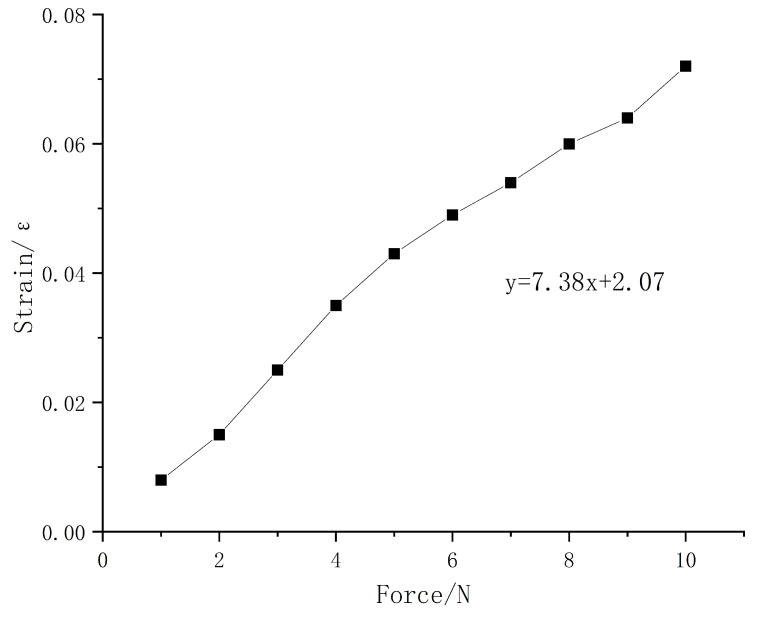
Pressure–strain simulation diagram.

**Figure 13:**
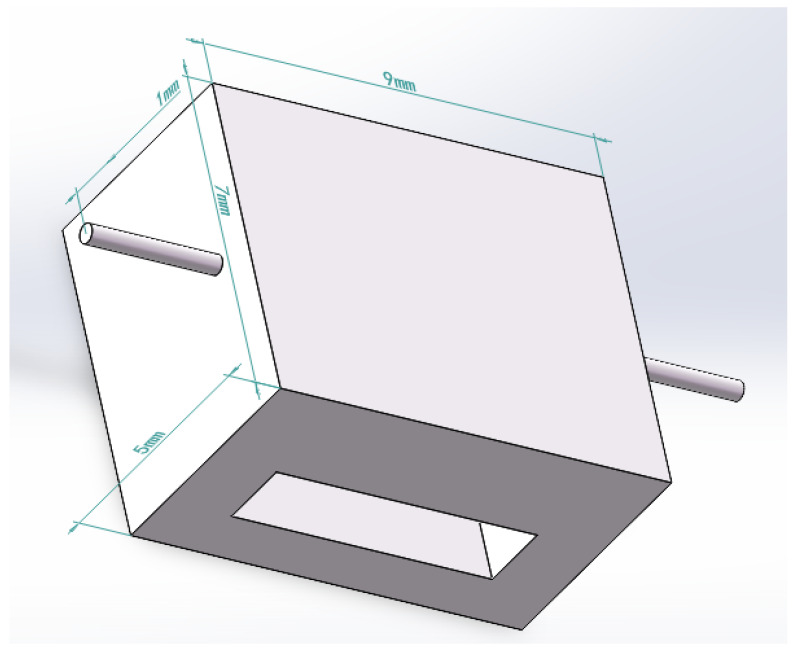
Model of the square hole sensing element.

**Figure 14:**
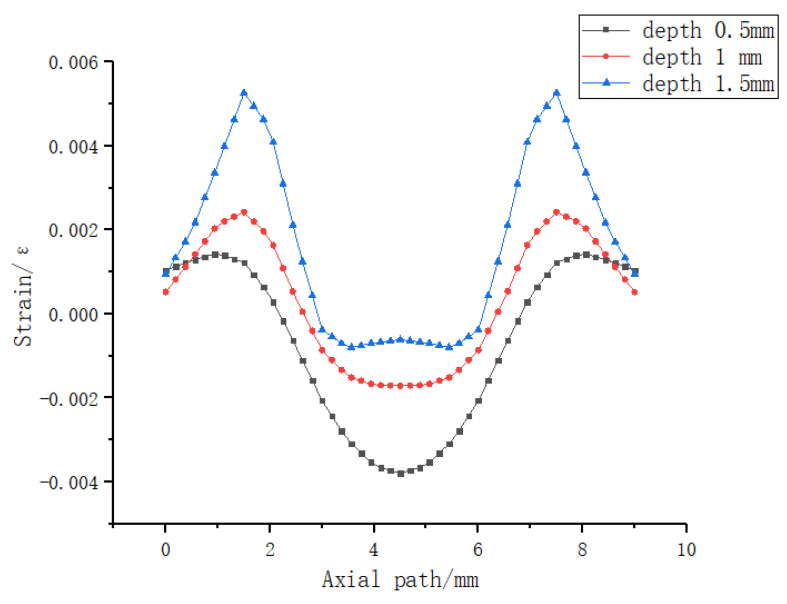
Strain diagram of fiber Bragg grating at different embedding depths under 2 N uniformly distributed load conditions.

Firstly, the solid sensing element and the square hole sensing element are simulated and compared:

a. Simulation analysis of the solid sensor element. The solid sensor element model is shown in Figure 10. The FBG is embedded in the center of the solid PDMS matrix, and the embedding depth of 1 mm is taken as an example for simulation analysis. A uniform load of 2 N is applied along the *z*-axis to the upper surface of the solid sensing element to obtain the axial strain of the optical fiber, as shown in Figure 11. As can be seen from Figure 11, when the PDMS elastomer encapsulated with FBG is subjected to external pressure, the internal fiber grating produces axial strain, resulting in the central wavelength shift of the grating. The strain on the axial path of the optical fiber is symmetrically distributed. The strain changes less toward the center, and the strain at the center reaches 1500 microstrain. The strain variation on the axial path is non-uniform. A uniform load of 1–10 N was applied to the solid structure, and the pressure–strain simulation diagram is shown in Figure 12. It can be seen from the figure that the sensitivity of the solid structure is 7.38 pm/N.

b. Simulation analysis of the square hole sensing element. As shown in Figure 13, the dimensions of the sensor element with holes are the same as those of the solid sensor element. A square hole structure was designed in the lower half of the model. The depth of the upper surface of the square hole is 2.5 mm, and the FBG is embedded above the square hole. A uniform load of 2 N is applied to the upper surface of the square hole sensing element in the vertical direction. According to the simulation analysis results in Figure 4, the best embedding depth of the 5 mm thick PDMS matrix is 1–2 mm from the upper surface. Therefore, 0.5 mm, 1 mm, and 1.5 mm were selected for comparative simulation analysis of different embedding depths, and the axial strain curve was obtained, as shown in Figure 14. The following conclusions were drawn from the simulation analysis: when the embedding depth of FBG is 0.5 mm, the overall variation value of strain in the central section of FBG is 700–1000 microstrain; when the embedding depth is 1 mm, the overall variation value of the grating section is 1870–2000, the fluctuation is small, and the strain value tends to be stable; and when the embedding depth of FBG is 1.5 mm, the overall variation value of strain in the central section of FBG is 2100–4000 microstrain. In conclusion, when the embedding depth is 1 mm, the overall change is controlled within 130 microstrains, and the fluctuation is small and can be maintained near the stable value, indicating that it has good strain uniformity. The strain value of the square hole structure is significantly higher than that of solid structure, which shows that the designed square hole structure can effectively increase the sensitivity of pressure perception.

In conclusion, through the comparative simulation analysis of the solid sensing element and square hole sensing element, it can be concluded that it is feasible to solve the problem of strain non-uniformity by using the newly designed sensing element with holes; the non-uniform strain on the grating path can be reduced by adjusting the grating embedding depth of the square hole.

**Figure 15:**
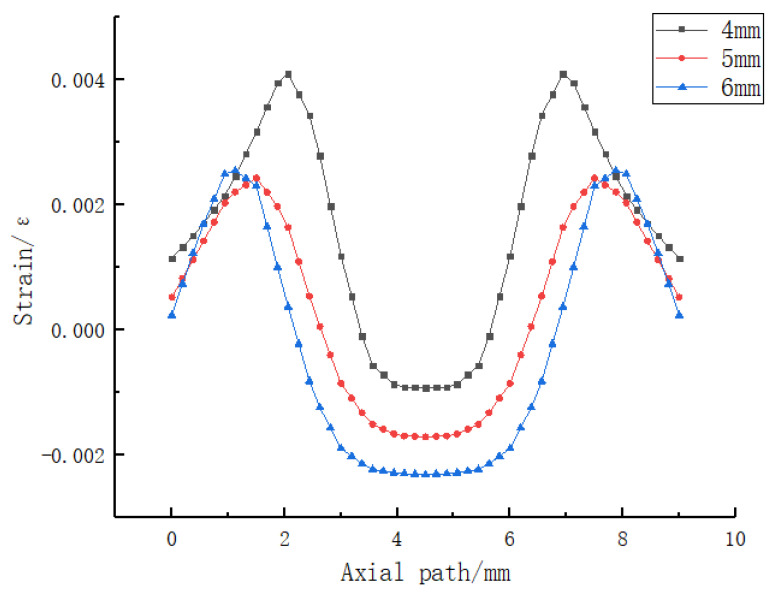
Strain diagrams of fiber Bragg gratings with different square hole sizes under 2 N uniformly distributed load conditions.

Secondly, a reasonable square hole section size was designed. As shown in Figure 15, 2 N uniformly distributed load was applied to the upper surface of the sensing element with holes, and the axial strain curves with different square hole lengths were compared. The following conclusions can be drawn from the simulation analysis:

a. After the simulation analysis of square hole structures with length of 4 mm, 5 mm, and 6 mm, the axial strain curves on the grating path were obtained;

b. The longer the square hole length is, the longer the uniform axial strain section on the grating path is;

c. When the length of square hole is 6 mm, the length of uniform strain section is 2.5 mm;

d. The longer the length of the square hole, the larger the average strain measured on the grating path;

e. The notable high degree of symmetry exhibited in Figure 14 and Figure 15 can be attributed to the symmetrical nature of both the sensor packaging and the applied loading conditions. Consequently, this symmetrical configuration led to highly symmetric strain results as well.

Therefore, the length of the square hole was chosen to be 6 mm, and, considering the stability of the structure under load conditions, the final size of the square hole section was designed to be 6 mm × 1.5 mm.

In summary, through the above simulation analysis, it was determined that the overall dimensions of the tactile sensing element are 7 mm × 9 mm × 5 mm, and it was proved that the square hole sensing element can effectively solve the problem of non-uniform strain. Finally, the embedding depth of FBG in the model is 1 mm, the embedding depth of square hole is 2.5 mm, and the section size of square hole is 6 mm × 1.5 mm.

## 4. Sensing Experiment and Analysis of FBG Pressure Tactile Element

### 4.1. Fabrication of FBG Pressure Tactile Sensing Element

**Figure 16:**
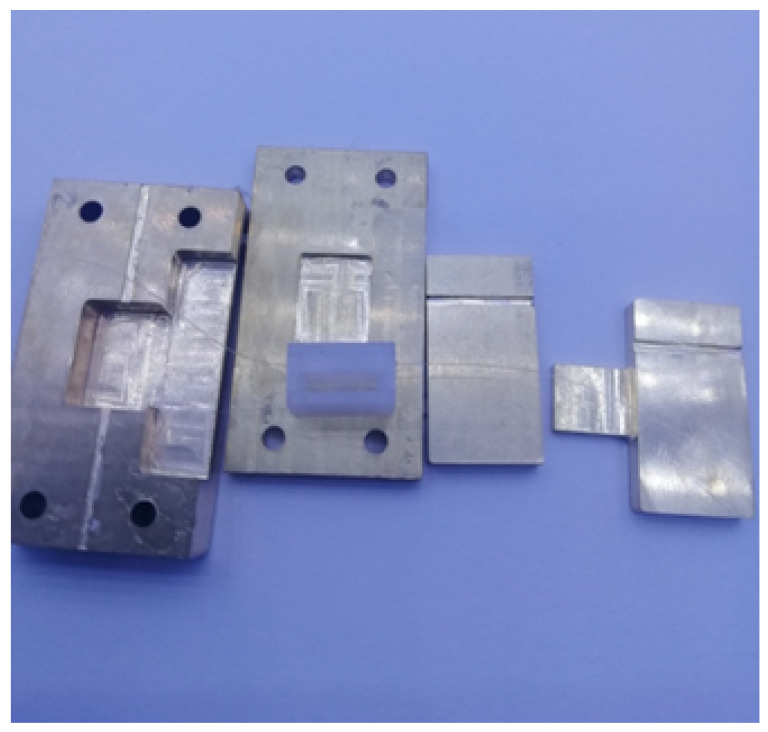
Sensor manufacturing mold.

In order to verify the performance of the model with holes, we made two types of sensing elements, which are the solid and the square hole structures. The mold was designed as a triple modular structure, as shown in Figure 16. In order to facilitate processing and demolding, the material of the mold was selected as copper. A groove was reserved at the center line of the lower mold cavity to fix the FBG, and a rectangular groove was added at the side of the cavity to pour the PDMS mixture. The upper mold retained a square groove with a depth of 1 mm, which corresponds to the requirement that the embedding depth of the FBG is 1 mm from the upper surface. The upper mold and the lower mold were fastened by bolts, and the movable block on the side was inserted in the form of a plug after injecting the PDMS mixture into the cavity. The protruding rectangular block formed a square hole in the cavity. By changing the rectangular block on the side, two models of the solid structure and square hole structure can be obtained. This form of mold is not only convenient for pouring and demolding but also can be used repeatedly.

When making the sensor, the PDMS glue and the curing agent were mixed at a ratio of 10:1, stirred evenly, and left to stand at room temperature for more than one hour to remove the microbubbles in the solution. The FBG was arranged in the groove in advance, the upper and lower molds were tightened with bolts, the PDMS mixture was poured slowly from the side, and then the movable block was pushed slowly, and the excess liquid was discharged from the drain hole. The movable block and the molds were locked with C-clip to ensure that the movable block did not slide. Finally, the completed pouring mold was placed into a drying oven for 130 min at a temperature of 75 ${}^{\circ }$C.

**Figure 17:**
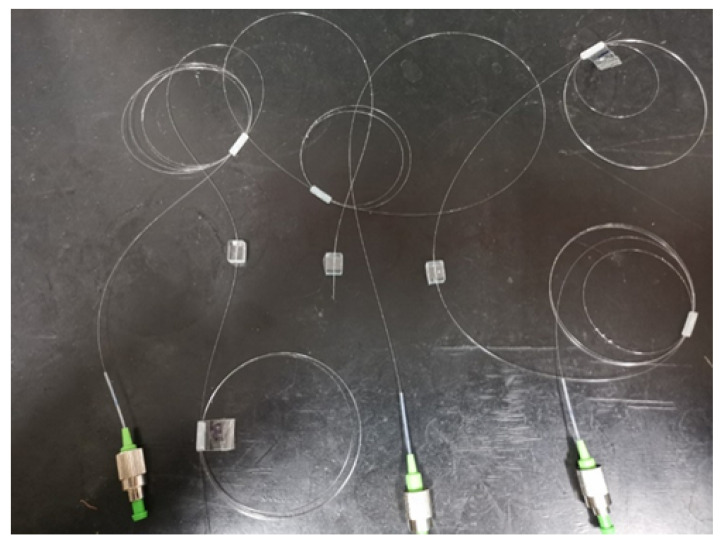
Prepared fiber grating tactile sensor.

According to this method, the solid and square hole sensing elements were fabricated, and the grating length is 2 mm. It can be seen from the simulation results in Figure 15 that there is a uniform axial strain of 2.5 mm at the center of the optical fiber. Therefore, a 3 mm grating was selected to make a square hole sensing element for comparison. The fabricated FBG tactile sensor is shown in Figure 17. The structure type, grating length, and initial grating center wavelength are shown in the following Table 1:

### 4.2. Pressure Tactile Sensing System

**Figure 18:**
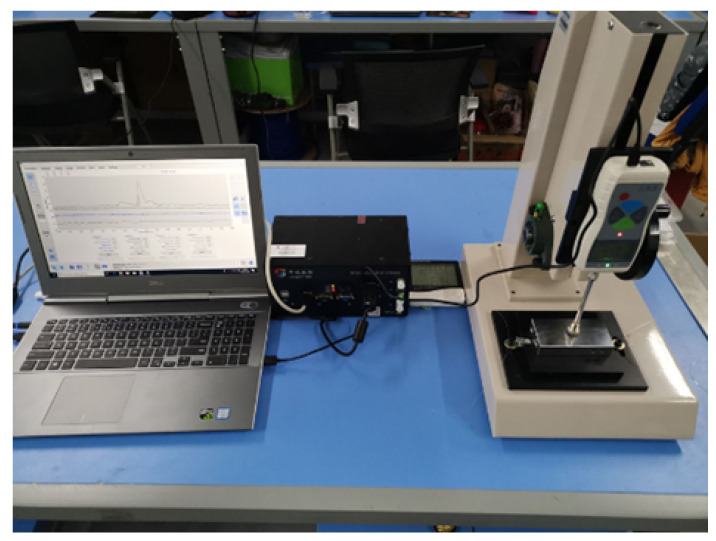
Fiber grating sensor detection system (including press, demodulator, PC, thermometer, and tactile sensor).

The tactile sensing detection system built in the experiment is composed of the FBG tactile sensor, press, demodulator, and computer, as shown in Figure 18. The selected sensors were produced by Teclico Optical Technology Co., Ltd. (Beijing, China), with a signal-to-noise ratio of 30 dB and reflectivity of 70%. The demodulator is the FSM04 soft tissue detection system produced by Zhongshi Wulian. The demodulator has 4 channels, a resolution of 0.1 pm, a range of 1510–1590 nm, a sampling frequency of 1000 Hz, and an internal light source and coupler. The loading press consists of two parts, including a screw support and a press. The resolution of the press can reach 0.001 N. In the process of tactile sensor loading, the software of the press can obtain the real-time magnitude of the loading force, and the computer can also measure the wavelength shift through the demodulator at the same time.

### 4.3. Experiment and Analysis of Pressure Tactile Sensing

During the experiment, the FBG tactile sensor was glued on a smooth platform. The press applied a uniform load on the upper surface of the sensor through the probe. By changing the stroke of the screw support, the applied load was controlled, and the center wavelength shift of the FBG was collected after the pressure was stable. For massage robots, the pressure on human skin is usually about 5 N, so the test pressure on tactile sensors should not be too large and should be controlled at 0–10 N.

1. Linearity experiment and analysis

**Figure 19:**
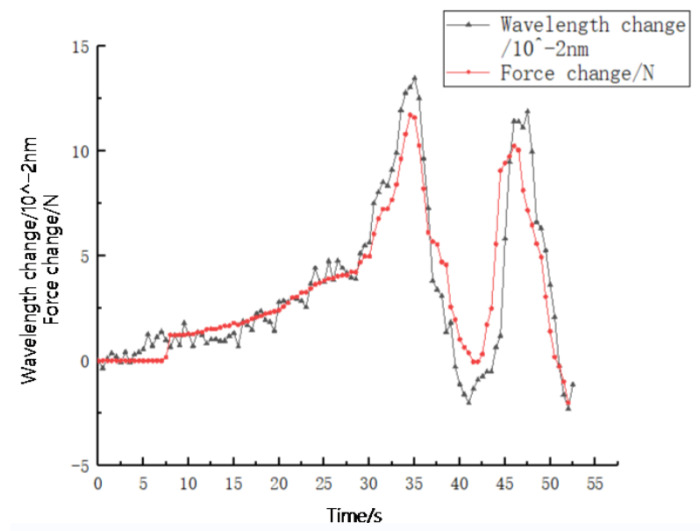
Sensor 1 pressure–wavelength change.

In order to control the influence of temperature on the central wavelength of FBG, the pressure contact experiment of the sensor was carried out at room temperature of 23 ${}^{\circ }$C. Taking sensor 1 as an example, the FBG tactile sensor was subjected to rapid and repeated loading–unloading by using a press. The pressure change curves obtained by the computer were compared with the center wavelength shift curves of the FBGs, as shown in Figure 19. In order to facilitate the comparison, the wavelength shift uses the order of $10^{-2}$ nm. It can be seen from the figure that the central wavelength shift curves of FBGs are consistent with the loading pressure curves, indicating that the FBG tactile sensor embedded in PDMS can better sense the change in external pressure.

**Figure 20:**
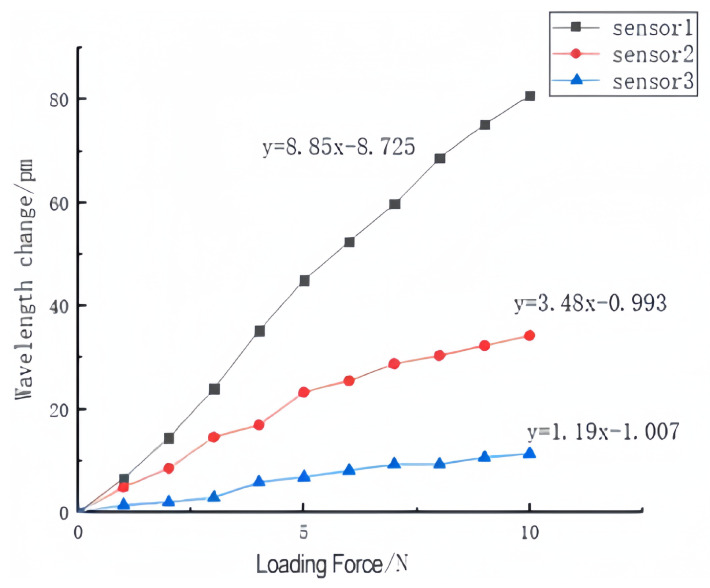
Center wavelength change in three sensors.

Three FBG tactile sensors were loaded from 0-10 N in steps of 1 N. The wavelength shift values of FBGs were recorded, and the curves for the central wavelength shift were fit, as shown in Figure 20. The following conclusions can be drawn from Figure 20:

a. The center wavelength shift values of the three sensors increased with the increase in the load, and tends to be linear;

b. The deformation of sensor 1 is stable, the linearity is the best, and the fitting degree reaches 0.995;

c. The linear fitting degree of the center wavelength shift of sensor 2 with grating length of 2 mm reaches 0.969, and that of sensor 3 with grating length of 3 mm reaches 0.971.

According to the curves of wavelength with pressure, the sensitivities of sensor 1, sensor 2 and sensor 3 are 8.85 pm/N, 3.48 pm/N, and 1.19 pm/N, respectively. Sensor 1 has a good resolution and can clearly distinguish the change of 0.1 N; considering the uniformity of strain, the linearity of sensor 2 is affected, and the resolution is 0.2 N. It can be seen from Figure 12 that the simulation sensitivity of the solid structure under the effect of external load is 7.38 pm/N, which is basically consistent with the 8.85 pm/N of the experimental results.

The experimental results show that:

a. All three sensors show good linearity;

b. Sensor 1 is a solid structure, which has more stable deformation and better linearity under loading conditions;

c. Sensor 2 adopts a 6 mm × 1.5 mm square hole structure with the grating length of 2 mm, which ensures the uniformity of axial strain, but its linearity is affected;

d. The linear fitting slope of sensor 3 is poor. The reason is that the increase in the grating length causes the two ends of the grating to not be uniformly strained, resulting in insensitivity to load changes;

e. The sensitivity of the three sensors is slightly lower than the simulation data. The possible reason is the fact that the FBG is not tensioned in advance in the manufacturing process.

2. Repeatability experiment and analysis

**Figure 21:**
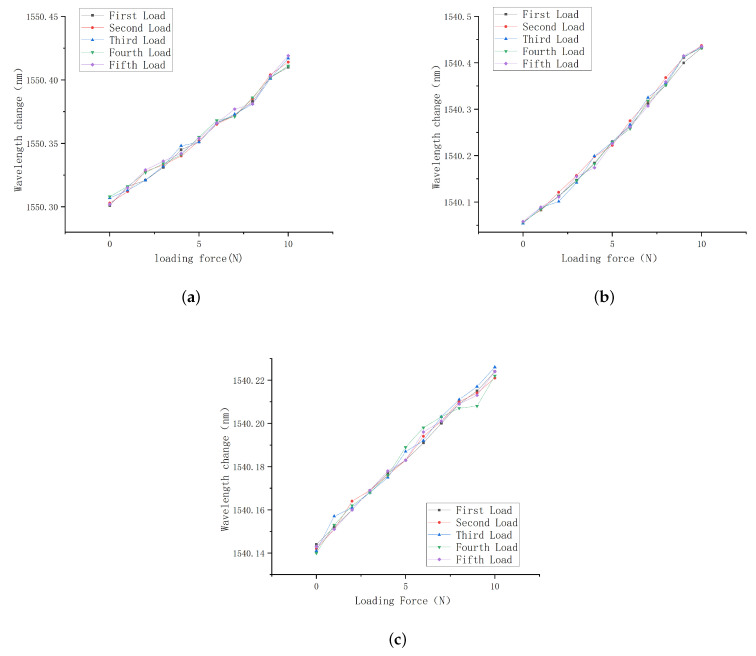
Characteristic curve for five loading. (**a**) Sensor 1. (**b**) Sensor 2. (**c**) Sensor 3.

The three sensors were loaded in steps of 0–10 N five times in steps of 1 N. The wavelength shift curves of each sensor are shown in Figure 21. As can be seen from the figure, the coincidence degree of the quintic loading curve of sensor 2 is the highest, the performance of sensor 1 is the second, and the five curves of sensor 3 are obviously separated.

The experimental results show that:

a. Sensor 1 and sensor 2 both using a 2 mm long grating show good repeatability;

b. The grating of sensor 3 is longer, which cannot guarantee the uniform strain of the whole grating, and its repeatability is poor;

c. The different repeatability displayed by sensors 1, 2, and 3 is also closely related to the sensitivity of the sensors. For sensors with high sensitivity, their linearity performance is also better.

3. Creep experiment and analysis

**Figure 22:**
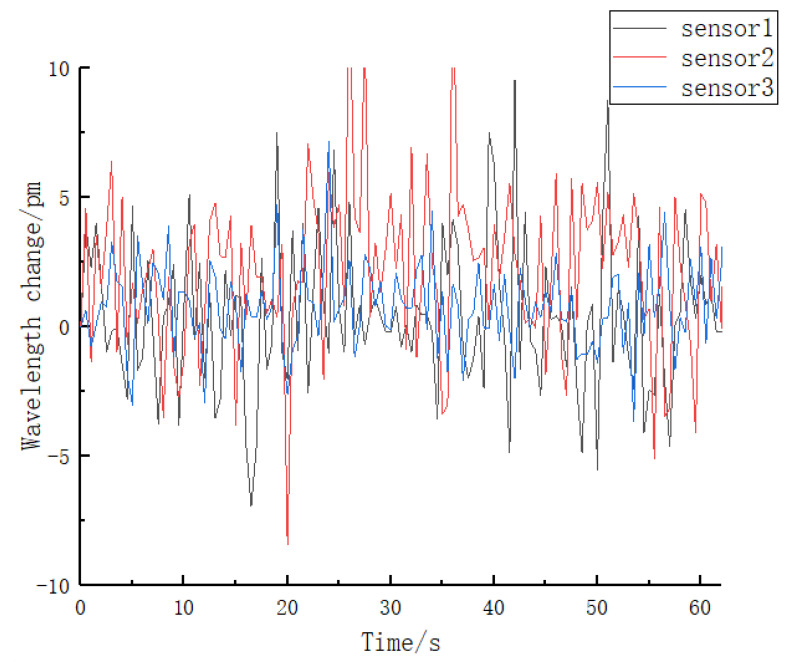
Characteristic curve for double loading.

The matrix of the tactile sensor made in this study is an elastomer structure. Creep is expressed as the central wavelength shift of FBG with time after the elastomer is stressed. The temperature was maintained constant while applying a pressure of 2 N to the upper surfaces of the three sensors individually for a duration exceeding one minute. Subsequently, the center wavelength shift of the FBG was recorded during this process. The resulting curves depicting the central wavelength shifts of the FBGs are visualized in Figure 22.

The experimental results indicate that, after a certain period of time under constant load conditions applied to the surface of the elastomer, the wavelength shifts of the three sensors exhibit fluctuations near the zero scale, thereby reflecting favorable creep properties of the elastomer. From the provided figure, it can be observed that sensors 1 and 2, which possess a grating length of 2 mm, exhibit a greater central wavelength shift compared to sensor 3 with a grating length of 3 mm. This observation suggests that an increase in grating length results in a smaller central wavelength shift of the fiber Bragg grating (FBG), indicating improved resistance to creep. These findings align with the theoretical expectations based on the relationship between grating length and the behavior of FBGs.

In conclusion, the above sensing experiments verify that the FBG sensor with holes designed in this study can effectively detect the tactile pressure and has good sensing performance.

## 5. Experiment and Analysis of Robot Finger Pressure Tactile Sensing

### 5.1. Robot Finger Pressure Tactile Sensing System

**Figure 23:**
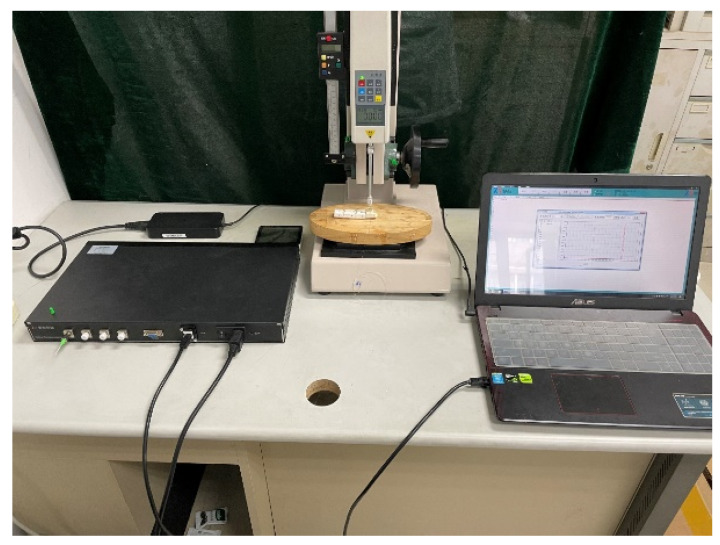
Pressure tactile experimental sensing system with tactile sensor pasted on the robot finger.

**Figure 24:**
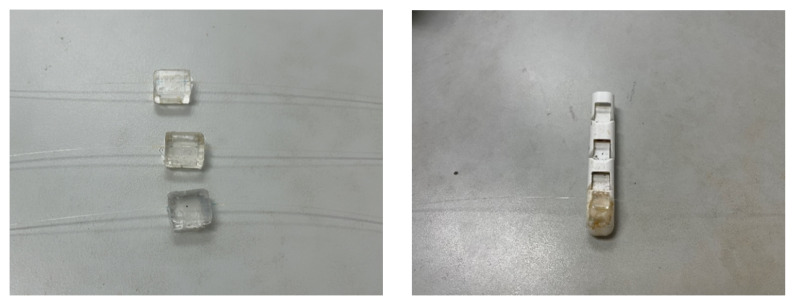
The three kinds of sensors.

The pressure tactile sensing experiment system of the robot finger is composed of the robot finger, the FBG tactile sensor, the press, the demodulator, and PC, as shown in Figure 23. The demodulator is the ZX-FP-C medium-speed instrument produced by Zhixing Technology. The demodulator has 4 channels, a resolution of 1 pm, a detection wavelength range of 1528–1568 nm, a sampling frequency of 1000 Hz, and an internal light source and coupler. The resolution of the press used in the experiment is 0.001 N. In the pressure tactile experiment, the PC can measure the central wavelength shift in real time through the demodulator. The sensor made by the mold is shown in Figure 24. The structure type, grating length, and initial grating center wavelength are shown in Table 2.

### 5.2. Experiment and Analysis of Robot Finger Pressure Tactile Sensation

1. Single-finger sensitivity experiment and analysis

**Figure 25:**
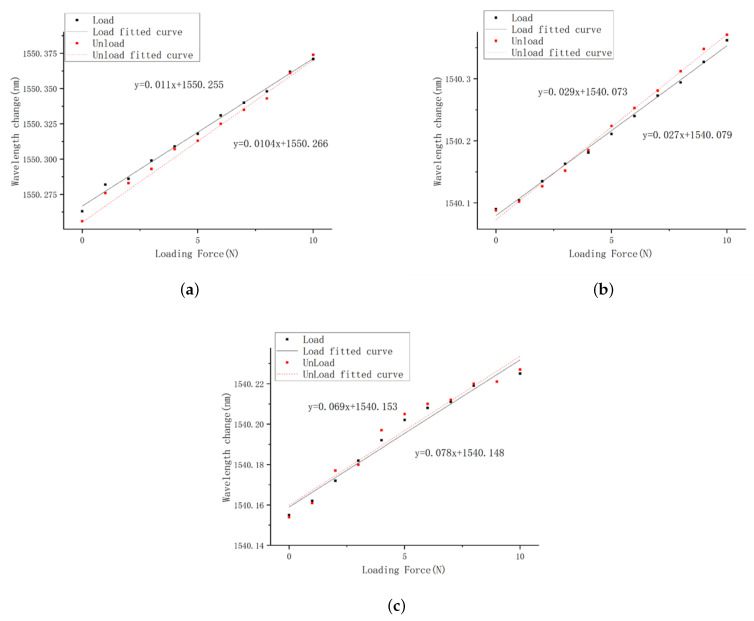
Center wavelength change graph of single-finger sensitivity. (**a**) Sensor 1. (**b**) Sensor 2. (**c**) Sensor 3.

The three sensing elements in Figure 24 were successively pasted on the first knuckle of the robot, and the pressure tactile experiments were carried out. The load–unload experiments were conducted on sensors through the press. The pressure range is 0–10 N in steps of 1 N. The central wavelength shift values of the sensor under different load conditions were recorded in real time using the PC, and the linear fitting was carried out. The central wavelength curves are shown in Figure 25.

The following conclusions can be drawn from Figure 25:

a. The central wavelength of the three sensors changes with the change in load, and the central wavelength shift tends to a linear distribution. The three sensors can identify the loading and unloading of external pressure;

b. The determination coefficients (R2) representing the goodness of fit were calculated for each of the three sensors during both the loading and unloading phases. For the loading phase, the obtained R2 values were 0.99616, 0.99599, and 0.96302 for the three sensors. Similarly, during the unloading phase, the corresponding R2 values for the same sensors were determined to be 0.99095, 0.99481, and 0.94388. The loading force sensitivities of the three sensors are 10.4 pm/N, 27.3 pm/N, and 6.9 pm/N, and the unloading force sensitivities are 11.4 pm/N, 29.8 pm/N, and 7.8 pm/N. The approximation errors of loading and unloading of sensors are (0.0021 nm and 0.0047 nm), (0.0048 nm and 0.0059 nm), and (0.0041 nm and 0.0068 nm). The linearity and sensitivity of loading and unloading are basically the same;

c. Sensor 2 has the highest loading and unloading sensitivity to external pressure. Compared with sensors 1 and 3, due to its internal square hole structure, it can enhance the tactile sensing to produce greater axial strain than other sensors under the effect of vertical load. At the same time, the grating length of 2 mm can maintain better uniformity of the axial strain during loading and unloading.

The experimental results show that the square hole structure has a sensitization in the finger pressure tactile experiment and can solve the problem of non-uniform strain. Among the three sensors, the grating length of 2 mm is the best choice.

2. Single-finger lateral force pressure experiment and analysis

**Figure 26:**
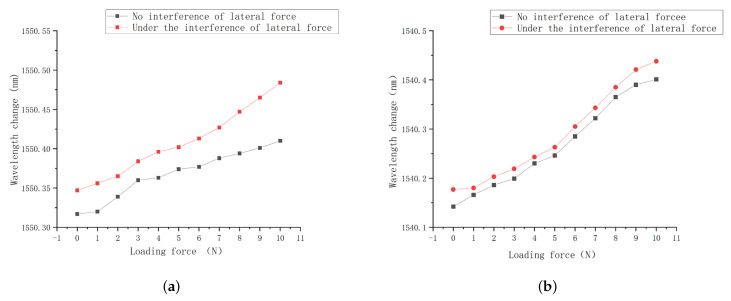
Pressure–center wavelength diagram. (**a**) Sensor 1. (**b**) Sensor 2.

In order to detect the interference degree of the square hole structure from the lateral force in the process of tactile sensing, sensor 2 was pasted on a single finger for the lateral force pressure experiment, and sensor 1 was selected for the comparison. The lateral force was controlled to 2 N constantly. The distributed loading experiment of 0–10 N was carried out for sensors 1 and 2, with a step of 1 N. The pressure center wavelength diagram is shown in Figure 26.

The following conclusions can be drawn from the figure:

a. Under the constant lateral force conditions, the sensors 1 and 2 tend to change linearly;

b. The center wavelength characteristic curve of sensor 2 has a high coincidence degree, and the wavelength deviation is controlled within 35 pm;

c. The coincidence degree of sensor 1 is low in lateral force interference comparison experiments, and the maximum deviation is 77 pm.

In order to further analyze the interference degree of lateral force on the sensing process, taking the wavelength value without lateral force as the reference value, the deviation degree of the wavelength change value under the lateral force conditions was calculated, and the average value of interference was calculated by averaging each interference rate.

The following conclusions can be drawn from the Table 3:

a. Under the influence of lateral force, the center wavelength shift values of sensors 1 and 2 under different load conditions undergo interference to a certain extent;

b. There is no vertical load at 0 N, and sensor 2 forms large strain under the effect of lateral force due to its high sensitivity, so the interference rate is higher than sensor 1;

c. Under the external load conditions, the interference rate of the central wavelength shift of sensor 2 by the lateral force is controlled within 10%, and the average interference value is 7.63%;

d. The interference value average of sensor 1 under lateral force conditions is 11.21%, which is higher than that of sensor 2. Sensor 1 is more vulnerable to the lateral force in the process of tactile sensing.

The experimental results show that the average interference of solid structure is 11.21%, and that of the square hole structure is 7.63%. In comparison, the square hole structure can ensure that the deviation between the wavelength value under the effect of lateral force and the wavelength value under the effect without lateral force is small, that it can more effectively reduce the interference of lateral force, and that the overall strain uniformity of the grating section under pressure also improves the measurement accuracy of robot finger pressure tactile perception.

## 6. Conclusions

Aiming at the problems of lateral force interference and non-uniform strain in the pressure tactile sensing of the robot finger, FBG was applied to the tactile sensor, and a sensor model with a square hole structure was designed in this study. Firstly, the optimal embedding depth of the sensor matrix model was determined by finite element simulation. Secondly, the simulation analysis of the pressure tactile sensor element based on the robot finger was carried out, and the square hole structure was designed to determine the element size and the square hole size. Finally, the FBG pressure tactile sensing system platform was built by using optical fiber sensing technology, and the sensors were made. Through the pressure tactile sensing system, the loading, repetition, and creep resistance of FBG tactile sensors, as well as the experiments of robot single-finger sensitivity and lateral force interference, were carried out. The results show that:

a. The designed tactile sensor has good linearity and repeatability, and the experimental results are consistent with the simulation results. The sensitivity of the sensor can reach 3.48 pm/N and has a good response to external loads. The loading characteristic curve has a high degree of coincidence, the deviation was controlled within 5 pm, and the overall change tends to be linear.

b. The designed tactile sensor has good creep resistance, and the wavelength shift of the sensor fluctuates around the zero scale, which is consistent with the grating length theory of FBG.

c. The resolution of the square hole structure of the designed pressure tactile sensor reaches 0.2 N, which is slightly lower than the 0.1 N resolution of the solid structure, but it can maintain good detection accuracy under the condition of reducing the interference of lateral force and meet the requirements of practical application.

d. In the robot single-finger experiment, the designed tactile sensor has a high fit with the robot finger, and the structure is relatively stable, which shows the feasibility of applying the pressure tactile sensor to the surface of the robot finger.

e. In the single-finger sensitivity experiment, the loading and unloading sensitivity of the pressure tactile sensor is basically the same. At the same time, the loading sensitivity and goodness of fit of sensor 2 are 27.3 pm/N and 0.996, respectively, and the values during unloading are 29.8 pm/N and 0.996, respectively. Compared with the solid structure, the sensing performance of the square hole structure is higher, which shows that this structure has a good sensitization on the tactile sensing of robot fingers and can effectively solve the problem of non-uniform strain in the sensing process.

f. In the process of tactile sensing, the average interference of the square hole structure is 7.63%, which is significantly lower than 11.21% of the solid structure, indicating that the sensor has good anti-lateral force performance and can effectively reduce the interference of lateral force.

The aim of this study was to integrate sensors onto robot fingers to furnish them with preliminary tactile monitoring capabilities. The subsequent stage involves the accomplishment of the tactile sensor’s objectives, including miniaturization, sensitization, and the implementation of wireless sensing functions.

Next, we will continue improving the work, add the sensor temperature compensation technology to eliminate the influence of temperature and improve the elastomer packaging process level, and compare and analyze with other different hole types and structures in order to enhance the sensor’s application performance in the field of pressure tactile intelligent sensing.

## Figures and Tables

**Table 1:** Parameters of fiber Bragg Tactile Sensor.

Sensor Number	Sensor Structure Type	Dimensions (mm)	Embedding Depth (mm)	Square Hole Embedding Depth (mm)	Grating Center Wavelength λ/nm	Grating Length *l*/mm
1	solid	7 × 9 × 5	1	-	1550.300	2
2	square hole	7 × 9 × 5	1	2.5	1540.100	2
3	square hole	7 × 9 × 5	1	2.5	1545.220	3

**Table 2:** Parameters of fiber Bragg tactile sensor.

Sensor Number	Sensor Structure Type	Grating Center Wavelength λ/nm	Grating Length *l*/mm
1	solid	1550.220	2
2	square hole	1540.060	2
3	square hole	1540.131	3

**Table 3:** Interference rate of sensors 1 and 2.

Sensor 2		Sensor 1	
**Pressure/N**	**Interference Rate/%**	**Pressure/N**	**Interference Rate/%**
0	24.6%	0	10.4%
1	8.43%	1	11.25%
2	9.13%	2	8.6%
3	10.05%	3	7.67%
4	5.65%	4	9.09%
5	6.91%	5	8.48%
6	7.01%	6	9.54%
7	6.52%	7	10.05%
8	5.48%	8	13.41%
9	7.95%	9	15.96%
10	9.23%	10	18.04%
Average interference value/%	7.63%	Average interference value/%	11.21%

## Data Availability

The data presented in this study are available on request from the corresponding author.
